# Genetic variation of *Echinococcus* spp. in yaks and sheep in the Tibet Autonomous Region of China based on mitochondrial DNA

**DOI:** 10.1186/s13071-019-3857-1

**Published:** 2019-12-27

**Authors:** John Asekhaen Ohiolei, Chen-Yang Xia, Li Li, Jian-Zhi Liu, Wen-Qiang Tang, Yan-Tao Wu, Danqulamu, Guo-Qiang Zhu, Bin Shi, Bao-Quan Fu, Hong Yin, Hong-Bin Yan, Wan-Zhong Jia

**Affiliations:** 1 State Key Laboratory of Veterinary Etiological Biology/National Professional Laboratory of Animal Hydatidosis, Key Laboratory of Veterinary Parasitology of Gansu Province/Lanzhou Veterinary Research Institute, CAAS, Lanzhou, 730046 Gansu People’s Republic of China; 2 Institute of Animal Science of Tibet Academy of Agricultural and Animal Husbandry Sciences, Lhasa, 854000 Tibet Autonomous Region People’s Republic of China

**Keywords:** *Echinococcus canadensis*, Haplotypes, Genetic variation, Tibet Autonomous Region

## Abstract

**Background:**

Cystic echinococcosis (CE) in humans and livestock is caused by *Echinococcus granulosus* (*sensu lato*). In China where CE is endemic, a number of studies have shown that *Echinococcus granulosus* (*sensu stricto*) is majorly responsible for CE. However, *E. canadensis* (G6) which is the second leading cause of CE is now being detected in most parts of the country. In this study, the species diversity and genetic variation of *Echinococcus granulosus* (*s.l.*) in four counties in Tibet Autonomous Region of China were investigated.

**Methods:**

Infection with *Echinococcus granulosus* (*s.s.*) in yaks and sheep was identified using NADH dehydrogenase subunit 1 and 5 (*nad*1 and *nad*5) mitochondrial genes while the genotype G6 of *E. canadensis* initially diagnosed with NADH dehydrogenase subunit 1 (*nad*1) was further confirmed by analysis of the complete mitochondrial genome and a phylogenetic network constructed based on the *nad*2 and *nad*5 genes.

**Results:**

Out of 85 hydatid cyst samples collected from slaughtered sheep (*n* = 54) and yaks (*n* = 31), 83 were identified as *E. granulosus* (*s.s.*) G1 (*n* = 77), G3 (*n* = 6) and 2 were identified as *E. canadensis* G6. Analysis of the *nad*1/*nad*5 genes revealed 16/17 mutations with 9/14 parsimony informative sites resulting in 15/14 haplotypes, respectively. Haplotype diversity (Hd) and nucleotide diversity (π) of *E. granulosus* (*s.s.*) population were 0.650 and 0.00127 for *nad*1 and 0.782 and 0.00306 for *nad*5, respectively, with an overall negative Tajima’s *D* and Fu’s Fs. A low F_ST_ indicated no genetic difference between isolates from sheep and yaks.

**Conclusion:**

Pockets of infection with *E. canadensis* (G6, G7, G8 and G10) have been previously reported in sheep, goats, yaks and/or humans in different parts of China. While the G6 genotype has been previously reported in sheep in the Tibet Autonomous Region, the detection in a yak in the present study represents the first to the best of our knowledge. Therefore, we recommend future surveys and control efforts to comprehensively investigate other potential intermediate hosts for the prevalence and genetic diversity of the *E. canadensis* group (G6, G7, G8 and G10) across the country and their inclusion into the existing CE control programme.
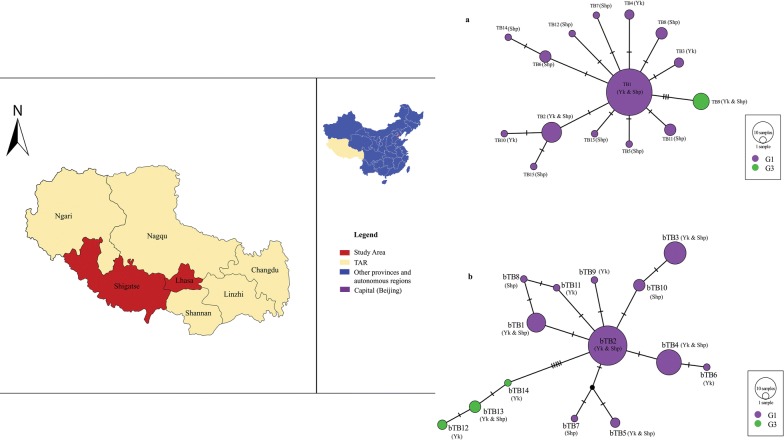

## Background

Cystic echinococcosis (CE) is a parasitic zoonosis caused by tapeworm species of *Echinococcus granulosus* (*s.l.*), which infect humans and livestock upon ingestion of the infective egg stage from the definitive host resulting in the development of hydatid cyst of the metacestode larval stages in the liver, lungs and sometimes in other organs. Within the *E. granulosus* complex, high genetic variation which affects species/genotype infectivity and preference to intermediate hosts has been recognised based on studies of parasite mitochondrial DNA [1–3]. So far, known species in this complex include *E. granulosus* (*sensu stricto*) (G1/G3), *E. equinus* (G4), *E. ortleppi* (G5), *E. canadensis* (G6, G7, G8 and G10) and *E. felidis* [4]. Meanwhile, the species status of the *E. canadensis* group is still under debate as suggestions have been made to reclassify the group into *E. intermedius* (G6/7), *E. borealis* (G8) and *E. canadensis* (G10) [5, 6]. Nonetheless, for the purpose of convenience, we refer to the group as *E. canadensis* all through the report.

CE poses a serious public health concern in China as a result of livestock infection, increasing morbidity in humans and the consequent economic impact due to the cost of diagnosis, control and treatment. The prevalence of CE in China varies with location with the Qinghai-Tibet Plateau being the most endemic region. In China, a number of molecular epidemiological studies have identified *E. granulosus* (*s.s.*) as the leading cause of cystic echinococcosis [7–12]. However, pockets of infection due to *E. canadensis* (G6, G7, G8 and G10) are now being reported [12–15]. Previously, infection with *E. canadensis* G6 genotype was frequently reported in the Xinjiang Uygur Autonomous Region [16, 17]. However, recent reports of the G6 genotype and other members of *E. canadensis* group in areas other than Xinjiang Uygur Autonomous Region, are raising concerns on its host range and public health significance [12, 14, 15, 18]. Therefore, it becomes imperative to update the status of CE and the genetic diversity of species especially in counties or communities in endemic regions, which will assist in monitoring the progress of ongoing CE management and control programmes across the country. In this study, the species diversity and genetic variation of *Echinococcus* species in four counties in the Tibet Autonomous Region of China were investigated and for the first time, the presence of *E. canadensis* (G6) in yak was confirmed using mitochondrial DNA markers (*nad*2 and *nad*5) and the complete mitochondrial genome analysis.

## Methods

### Study area

**Fig. 1:**
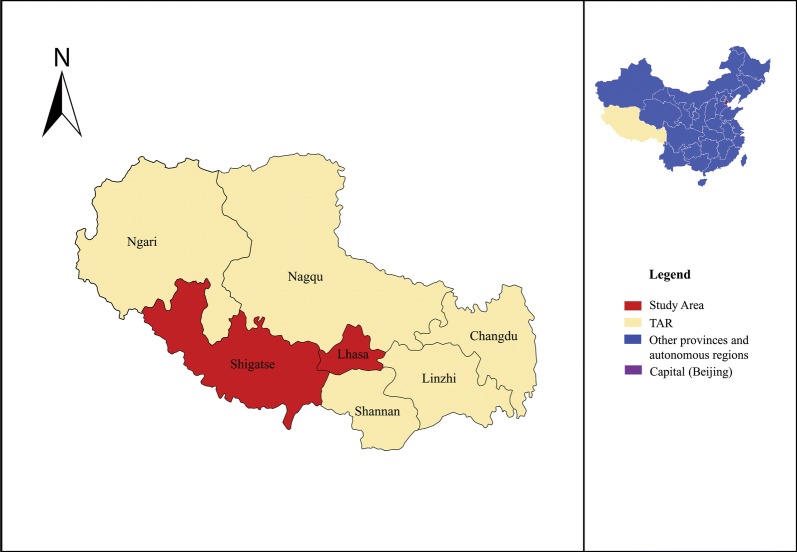
Map of the Tibet Autonomous Region of China showing the study area

The Tibet Autonomous Region is a plateau with grassland vegetation. It has an average elevation of about 4000 m and covers a landmass of about 1.23 million km^2^ bordering Qinghai Province and Xinjiang Uygur Autonomous Region to the north, Sichuan and Yunnan provinces to the east, and to the south, Myanmar, India, Bhutan and Nepal. Tibetans live mostly as seminomadic pastoralists with goats, sheep, yaks, horses and dogs as their main domestic animals. The Tibet region has a total of 74 counties in 7 prefectures that include 692 townships and 5260 villages with a total population of 3 million [19]. The study was carried out in four counties in the region namely: Angren, Saga and Zhongba (Shigatse Prefecture), and Dangxiong (Lhasa City) (Fig. 1).

### Sample collection and DNA extraction

Between October and December 2017, a total of 85 hydatid cysts were collected from the lungs and liver of slaughtered sheep (age 1–11 years) and yaks (age 5–18 years) during routine meat inspection in slaughterhouses and slabs in the counties. The slaughtered animals were raised in an extensive management system (free grazing) in the same counties where the study was conducted. The collected hydatid cyst samples (Additional file 1: Figure S1, Additional file 2: Figure S2) were then cleaned with 75% alcohol before the germinal layers were removed and washed repeatedly with phosphate buffer solution (PBS) and then transported in an ice box to the laboratory where they were stored at − 80 °C prior to DNA extraction. Further, a piece of the germinal layer excised from each hydatid cyst was crushed in liquid nitrogen before genomic DNA extraction was carried out using Qiagen Blood and Tissue DNA Extraction Kit (Qiagen, Hilden, Germany) according to the manufacturer’s instructions.

### DNA amplification and sequencing

PCR was performed in a 25 µl final reaction mixture containing 12.5 μl Premix *Taq*™ (Takara Bio, Kusatsu, Japan), 0.5 μl of each primer (10 pmol), 0.5 μl of the genomic DNA (*c*. 10–180 ng), and 11 µl RNAse-free water. The PCR conditions were as follows: initial denaturation at 95 °C for 5 min; 35 cycles of denaturation at 95 °C for 30 s, annealing at 55 °C for 40 s and elongation at 72 °C for 60–90 s; and a final extension step at 72 °C for 10 min.

The complete mitochondrial NADH dehydrogenase subunit 1 gene (*nad*1, 894 bp) was initially amplified for all isolates using the forward (5ʹ-ATT ATA GAA AAT TTT CGT TTT ACA CGC-3ʹ) and reverse (5ʹ-ATT CAC AAT TTA CTA TAT CAA AGT AAC C-3ʹ) primers [15]. In order to distinguish between genotypes of *E. granulosus* (*s.s*.) a primer pair amplifying a fragment of the *nad*5 gene (about 680 bp) reported to have conserved regions capable of discriminating G1 from G3 [20] was used, while the complete mitochondrial genome was amplified to correctly place the G6 genotype using previously designed primers [15] and newly designed complementary primers (Additional file 3: Table S1). Each PCR reaction yielded a single band detected in a 1.5% (w/v) agarose gel stained with GelRed^®^ (Biotium, Fremont, USA). Five microliters of the amplicon were used for visualization while the rest were used for sequencing in an ABI3730Xl DNA Analyser (Tsinqke Biological Technology, Beijing, China).

### Molecular and phylogenetic analysis

Following sequencing, the nucleotide sequences were viewed and manually corrected for any misread nucleotide by comparing the chromatogram file with the resulting sequences using Unipro UGENE v1.29.0 software and BioEdit [21]. The sequences of the complete mitochondrial genome were assembled stepwise with the help of DNAStar v7.1 program, BioEdit, and Unipro UGENE v1.29.0 software while making sure that the overlap sequences were identical. The final sequences were aligned with BioEdit [21]. The protein-encoding genes and rRNAs of the complete mitochondrial genome were annotated using GeSeq [22]; tRNAs were identified using tRNAscan-SE v2.0.3 and/or by a BLAST search against an *E. canadensis* genotype G6 mitochondrial genome sequence available in GenBank (GenBank: AB208063). The identity of the isolates *via* their nucleotide sequences was assessed using the NCBI BLAST program and compared with existing sequences in the GenBank database (https://blast.ncbi.nlm.nih.gov/Blast.cgi).

The population diversity indices were estimated in DnaSP v6 [23]. Median-joining networks for *E. granulosus* (*s.s*.) and *E. canadensis* G6 were constructed based on the nucleotide sequences of the mitochondrial *nad*5 [20] and concatenated *nad*2-*nad*5 [24] genes, respectively using PopART software [25]. Using DnaSP v6 [23], the population neutrality indices, Tajima’s D [26] and Fu’s Fs [27], and pairwise fixation index (F_ST_), were calculated. A Bayesian phylogenetic tree for *E. canadensis* G6 genotype was infered based on the concatenation of the 12 protein coding genes using MrBayes v.3.1.2 with a Markov Chain Monte Carlo (MCMC) sampling to assess the posterior distribution of parameters using a chain length of 4,000,000 states with 10% discarded as ‘burn-in’. The parameters were logged every 1000 states. Convergence was assessed by ensuring that the potential scale reduction factor (PSRF) approaches 1 [28] while the resulting tree was displayed by TreeView (http://taxonomy.zoology.gla.ac.uk/rod/treeview.html).

## Results

Out of the 85 isolates (54 from sheep and 31 from yaks) (Table 1), 83 were identified as *E. granulosus* (*s.s.*) comprising genotypes G1 (*n* = 77) and G3 (*n* = 6) and 2 (from a yak and sheep) were identified as *E. canadensis* (genotype G6) with > 99% identity to deposited sequences in GenBank. The *E. granulosus* (*s.s*.) *nad*1 gene sequences had a total of 16 polymorphic sites with 9 parsimony informative sites, while *nad*5 had a total of 17 mutations with 14 parsimony informative sites without indels. The observed nucleotide mutations also resulted in amino acid changes. Based on the *nad*5 (680 bp) gene, amino acid substitution between genotype G1 and G3 were observed at three positions as follows: 97, S to T; 105, I to V; and 219, G to S (Additional file 3: Tables S2, S3).

**Table 1:** Number of isolates analysed from all four counties in the Tibet Autonomous Region of China (TAR)

Location	Isolate/host		Total
	Sheep	Yak	
Angren county	–	1	1
Dangxiong county	1	–	1
Saga county	14	19	33
Zhongba county	39	11	50
Total	54	31	85

### Haplotype networks of *Echinococcus granulosus* (*s.s.*)

**Fig. 2:**
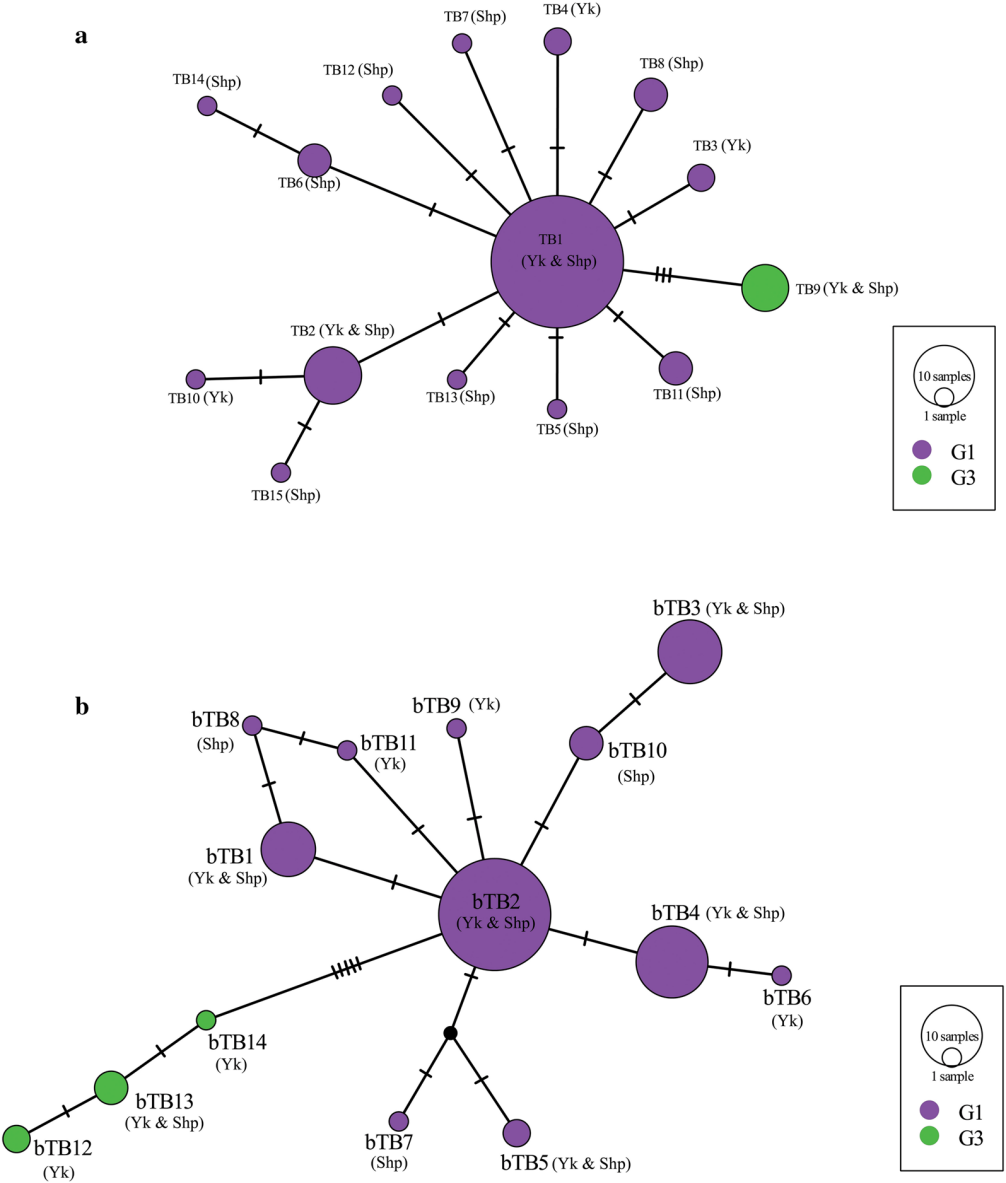
Median-joining networks of Tibetan population of 83 *Echinococcus granulosus* (*s.s.*) isolates (yak, *n* = 30, sheep, *n* = 53) **a** *nad*1 (894 bp) and **b** *nad*5 (680 bp). Circle sizes are proportional to the corresponding haplotype frequencies. Hatch marks represent the number of mutations

Of the 83 *E. granulosus* (*s.s.*) isolates, 15 haplotypes resulted from the analysis of *nad*1 gene sequences (Fig. 2a, Table 2) and 14 from *nad*5 gene (Fig. 2b, Table 2). The *nad*1 and *nad*5 networks formed a star-like configuration with a central haplotype TB1 and bTB2, respectively, constituting 57.8% and 41%, respectively, of the total *E. gransuslosus* (*s.s.*) population; these were separated from the remaining haplotypes by 1–3 (*nad*1) and 1–7 (*nad*5) mutational steps (Fig. 2a, b). The main haplotype in the *nad*1 network was found in 3 out of 4 of the investigated counties (Zhongba, Saga and Dangxiong) while the *nad*5 main haplotype was found in 2 (Zhongba and Saga) of the counties. In addition, all six G3 isolates based on the *nad*1 gene formed a single haplotype (TB9) with three mutational steps from the founder haplotype (TB1) and haplotypes bTB12, bTB13 and bTB14 based on the *nad*5 gene had 5–7 mutation steps from the main haplotype (bTB2) (Fig. 2a, b).

**Table 2:** *Echinococcus granulosus* (*s.s.*) haplotypes showing host, origin and their corresponding GenBank accession numbers

*nad*1					*nad*5				
Haplotype	Host	Origin	Freq.	GenBank ID	Haplotype	Host	Origin	Freq.	GenBank ID
TB1	Sheep and Yak	Zhongba, Saga, Dangxiong	48	MN269986	bTB1	Sheep and Yak	Zhongba, Saga	8	MN270001
TB2	Sheep and Yak	Zhongba, Saga	9	MN269987	bTB2	Sheep and Yak	Zhongba, Saga	34	MN270002
TB3	Yak	Zhongba	2	MN269988	bTB3	Sheep and Yak	Zhongba, Dangxiong	11	MN270003
TB4	Yak	Saga	2	MN269989	bTB4	Sheep and Yak	Zhongba, Saga	14	MN270004
TB5	Sheep	Zhongba	1	MN269990	bTB5	Sheep and Yak	Zhongba	2	MN270005
TB6	Sheep	Zhongba, Saga	3	MN269991	bTB6	Yak	Zhongba	1	MN270006
TB7	Sheep	Saga	1	MN269992	bTB7	Sheep	Zhongba	1	MN270007
TB8	Sheep	Zhongba, Saga	3	MN269993	bTB8	Sheep	Zhongba	1	MN270008
TB9	Sheep and Yak	Zhongba, Saga	6	MN269994	bTB9	Yak	Saga	1	MN270009
TB10	Yak	Zhongba	1	MN269995	bTB10	Sheep	Zhongba, Saga	3	MN270010
TB11	Sheep	Zhongba	3	MN269996	bTB11	Yak	Saga	1	MN270011
TB12	Sheep	Zhongba	1	MN269997	bTB12	Yak	Saga	2	MN270012
TB13	Sheep	Saga	1	MN269998	bTB13	Sheep and Yak	Zhongba	3	MN270013
TB14	Sheep	Saga	1	MN269999	bTB14	Yak	Zhongba	1	MN270014
TB15	Sheep	Zhongba	1	MN270000					

The diversity and neutrality indices of *E. granulosus* (*s.s.*) were calculated based on the sequences of *nad*1 and *nad*5 and indicated population expansion (Table 3). The pairwise fixation (F_ST_) value between isolates from both host populations was low (*nad*1 = 0.04925; *nad*5 = 0.06644) suggesting the lack of genetic differentiation.

**Table 3:** Diversity and neutrality indices for *Echinococcus granulosus* (*s.s.*) populations from Tibet Autonomous Region of China (TAR)

Index	*nad*1 (894 bp)			*nad*5 (683 bp)		
	Yak	Sheep	Overall	Yak	Sheep	Overall
No. of isolates	30	53	83	30	53	83
No. of mutations	7	13	16	16	14	17
Parsimony informative site	6	4	9	9	5	14
No. of haplotypes	6	12	15	11	9	14
Haplotype diversity (Hd)	0.618	0.663	0.650	0.818	0.755	0.782
Nucleotide diversity (π)	0.00154	0.00107	0.00127	0.00411	0.00230	0.00306
Tajima’s *D*	− 0.660	− 1.973*	− 1.851*	− 1.03778	− 1.475	− 1.119
Fu’s Fs	− 0.715	− 8.472*	− 10.098*	− 2.589	− 2.013	− 3.922

* Significant *P-*value

Haplotypes representing nucleotide sequences of *E. granulosus* (*s.s.*) *nad*1 and *nad*5 genes have been deposited in GenBank under accession numbers MN269986-MN270000 (*nad*1) and MN270001-MN270014 (*nad*5).

### *Echinococcus canadensis* phylogeny and network analysis

**Fig. 3:**
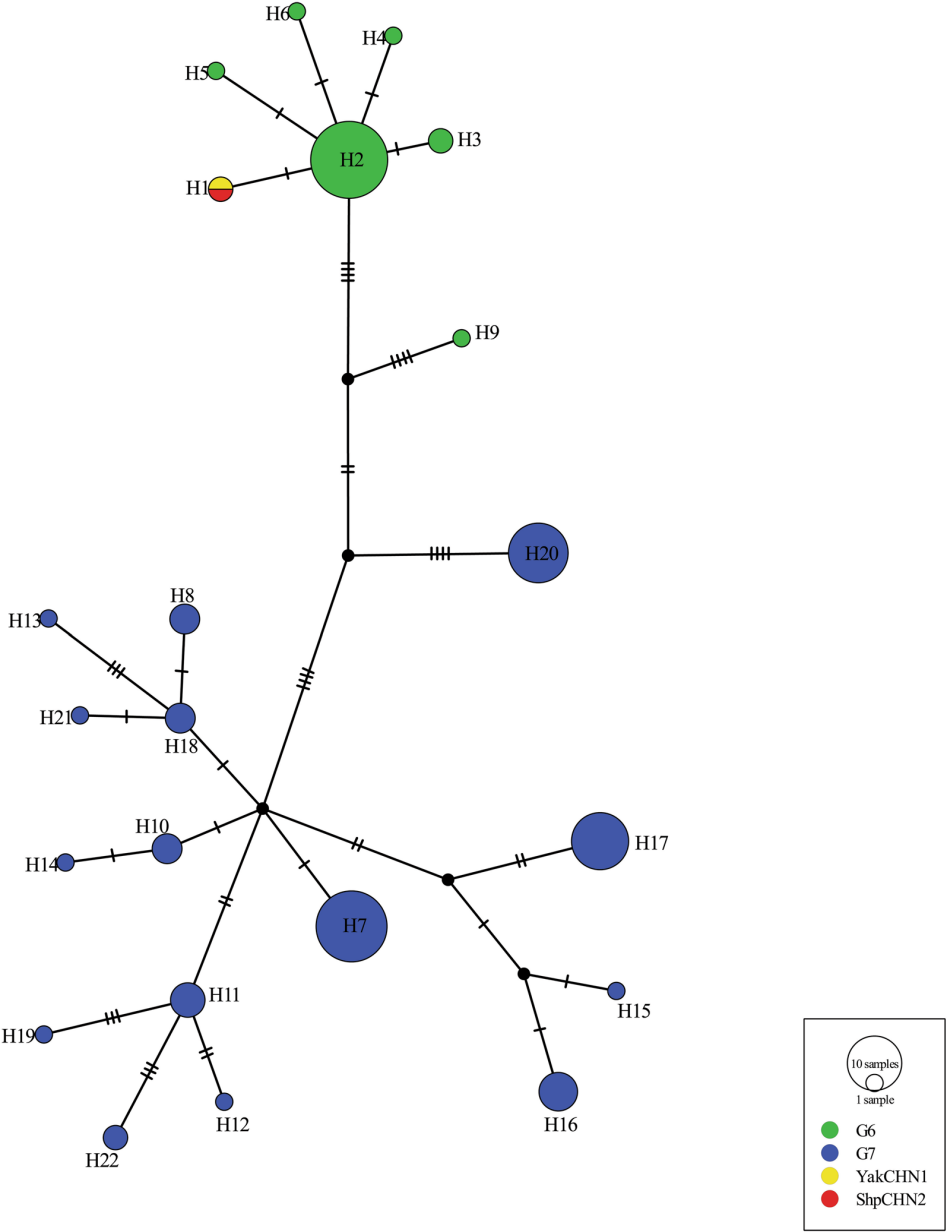
Median-joining network of concatenated *nad*2-*nad*5 (2454 bp) genes of Tibetan *Echinococcus canadensis* G6 isolates. Hatch marks represent the number of mutations. *E. canadensis* G6/G7 GenBank reference sequences MH300930-MH300954, MH300971 (*Gmon*) and MH300956-MH300970, MH300972-MH301022 (G7) were obtained from Laurimae et al. [28]. H9 represent the *Gmon* isolate from Mongolia whose phylogenetic relationship with the G6 and G7 genotypes and genetic identity remain unclear

The *E. canadensis* (G6) was detected in a sheep and yak from Zhongba (isolated from the liver) and Angren (isolated from the lungs) counties, respectively based on an initial analysis of the *nad*1 gene. Further BLAST query of the complete mitochondrial genome sequences (13,731 bp) gave a highest percentage coverage and similarity of 100% and 99.86% with genotype G6 (GenBank: AB208063) and 100% and 99.58% with genotype G7 (GenBank: AB235847), respectively. The genotype status was further confirmed by a median-joining network constructed by the *nad*2-*nad*5 gene sequences which provided a clear distinction from reference G7 nucleotide sequences (Fig. 3). The Bayesian phylogeny based on the 12 protein coding regions showed the G6 isolates from this study in the same cluster with other G6 sequences (Additional file 4: Figure S3). The reference G6/G7 sequences used in this analysis were from Laurimae et al. [29]. The resulting mitochondrial genome sequences of *E. canadensis* (G6) from this study were submitted to the GenBank database under the accession numbers MN340038 (YakCHN1) and MN340039 (ShpCHN2).

## Discussion

The Tibet Autonomous Region is one of the most CE endemic regions in the country. Previous reports have found prevalence to reach up to 10% in humans and 82% in intermediate hosts [30]. However, recent estimates have shown a huge decrease in the prevalence of CE with an average of 1.66% in humans and 11.84% in livestock [19]. In the region, CE transmission is usually maintained by the pastoral lifestyle of the inhabitants including the use of guard dogs which play a significant role in the transmission pattern through faecal contamination of the environment.

To date, a number of investigations have shed light on the genetic diversity within the genus *Echinococcus* and the intraspecific variation among *E. granulosus* (*s.s.*) using different DNA markers [1, 2, 31, 32]. In the present study, using mitochondrial DNA, the results lend support to previous observation of *E. granulosus* (*s.s.*) (G1) as the leading cause of CE in the Tibetan plateau indicating that of all species/genotypes of *Echinococcus* responsible for CE, this highly zoonotic group predominates in China [7–12].

Overall, the genetic variation observed among *E. granulosus* (*s.s.*) isolates and the results of neutrality indices as supported by the significant negative values of both Tajimaʼs *D* and Fu’s Fs indicated a negative selection or population expansion, a common feature of *Echinococcus* spp. population in the Tibetan plateau [10, 13, 31]. The low nucleotide diversity among *E. granulosus* (*s.s.*) in the present study has also been previously observed in the Qinghai-Tibet region and other parts of China [13, 31] as well as from outside China [32, 33], supporting the demographical expansion of the group. Also, the low F_ST_ value between the intermediate host populations suggests that both populations are genetically undifferentiated, indicating gene flow and alleles sharing between the subpopulations of *E. granulosus* (*s.s.*) and possibly with the overall population in the region which is plausible considering that both intermediate hosts utilized the same environment with no geographical barrier and are exposed to similar risk factors like contamination of pastures with faeces of infected dogs. The onset implication of gene flow on the parasite population includes increase genetic variation and host adaptability which over time, in the absence of natural selection and/or genetic drift, may reach gene homogeneity due to allele frequencies reaching equilibrium values and thus the appearance of a population structure characterised by a founder haplotype that is common to different host populations as seen in the present study. Similar observations of gene flow and the lack of genetic differentiation among populations of *E. granulosus* (*s.s.*) has also been reported in the Qinghai-Tibet region [10, 13, 31, 34] and other locations [32, 35].

The main haplotype of *E. granulosus* (*s.s.*), TB1, based on the *nad*1 network showed a 100% homology to Tunisian, Spanish, Turkish and Chinese isolates from sheep, Chilean isolate from cattle, and human isolates from Algeria and Finland [10, 36]. Similarly, the *nad*5 main haplotype also showed 100% similarity to GenBank sequences including Australian G1 isolate from sheep [37], Tunisian and Algerian human G1 isolates and Chilean cattle isolates [36]. This observation supports the diffusion theory of *E. granulosus* (*s.s.*) group from the Middle East where it is believed to have originated as a result of sheep domestication and the subsequent dispersal of the tapeworm to Europe, Africa, the Americas and Asia by the anthropogenic movement of intermediate host animals through livestock trade [12, 38].

Differences in genetic variation as reflected in haplotype distribution among different populations of *E. granulosus* (*s.s*.) in the Tibetan Plateau and other parts of China have also been observed [11, 31]. However, these differences could be a result of the short gene sequences analysed [39], the mitochondrial region investigated or the dissimilarities in the evolution rate of the various mitochondrial genes [13]. For instance, 83 isolates in this study, resulted in 15 haplotypes based on the complete mitochondrial *nad*1 gene while the partial *nad*5 gene resulted in 14 and are incomparable to the 28 haplotypes from 84 *E. granulosus* (*s.s.*) isolates from the Tibetan Plateau based on a concatenated sequence of *nad*1 and *atp*6 genes [10], 19 haplotypes reported by Wang et al. [13], using mitochondrial *nad*2 gene as well as 10 haplotypes among 45 isolates from humans, sheep, and yaks in western China based on mitochondrial *cytb* gene [11]. Nonetheless, these reports support the existence of intraspecific variation within *E. granulosus* (*s.s.*) and an expanding population in the region.

The population network of *E. granulosus* (*s.s.*) in this region as depicted by the median-joining network was characterised by a star-like configuration with a centrally placed haplotype similar to previous observations [12, 13, 31]. Meanwhile, a study observed that the phylogenetic network of *E. granulosus* (*s.s.*) in the Tibetan region [12] differs considerably with that of the Middle East [39, 40] where *E. granulosus* (*s.s.*) is also prevalent [32, 39, 41] such that the Tibetan phylogenetic network is characteristically star-shaped with a centrally placed haplotype and low genetic variation in contrast to that of the Middle East but was found to be somewhat similar to the radial phylogenetic network reported in Africa [32]. This pattern of network is also characteristic of an expanding population and again strengthens the diffusion theory of the group from the Middle East. Conversely, recent studies based on the analysis of near-complete mitochondrial genomes of a large dataset of *E. granulosus* (*s.s.*) isolates from different hosts/regions suggested the absence of a central haplotype [36, 42]. However, the observation of a central haplotype in the present study and in most studies could be a geographical limitation or a result of short DNA fragment analysed.

The *E. canadensis* G6 isolates from this study on comparison with the available partial gene sequences of *E. canadensis* G6 and G7 from the Tibetan Plateau region revealed some differences involving nucleotide base substitutions. For instance, a comparison of the partial *nad*1 gene sequences of *E. canadensis* identified as G7 from goat in Qinghai (GenBank: JQ317986) with our *nad*1 gene sequences showed changes at position 477 (C/T). Similarly, changes at positions 717 (T/C), 723 (A/T), 1122 (A/G) and 1125 (A/G) were also observed with the partial *cox*1 gene sequences of *E. canadensis* G6 genotype from a human in Xinjiang (GenBank: DQ356884). Furthermore, comparison of the complete *cox*2 gene sequences from this study with the partial *cox*2 gene sequences of the G6 genotype previously isolated from sheep in the Tibet Autonomous Region (GenBank: KC692991, KC692992) showed similar nucleotide sequences without variation. However, the G6 genotype from this study, confirmed by comparing the complete mitochondrial genome sequences with reference G6 and G7 genotypes available in GenBank and the phylogenetic network of the *nad*2-*nad*5 gene sequences [24], possessed the G6 peculiar nucleotide bases at the defining positions for *E. granulosus* (*s.l.*) genotypes G6 and G7 as previously described [24].

The presence of the highly zoonotic *E. granulosus* (*s.s.*) in China remains a serious public health concern as the country accounts for over 90% of the global human CE burden [43]. More so, the growing evidence of human and livestock infection with members of the *E. canadensis* group raises more public health concerns, suggesting the need for improved surveillance in order to appraise the level of infection due to members of this group. Although integrated measures which include control of the source of infection by deworming of dogs with anthelmintics, strict management practices in slaughterhouses and quarantine of livestock to restrict dogs from feeding on offal of infected animals, health education on the need for improved personal hygiene, and treatment of patients, are in place to mitigate CE transmission and to achieve control. However, more still needs to be done in the area of surveillance and appraisal of the control efforts in order to achieve control/eradication. Also, in the event of the use of vaccines, it is important to understand the species/genetic diversity of *Echinococcus* in an endemic area as immune response may differ due to infection by different genotypes which consequently affect control efforts. For example, the EG95 orthologue in *E. canadensis* and *E. granulosus* (G1) has been found to differ in their amino acid constitution which could affect their immunogenic properties [44].

## Conclusions

Although *E. canadensis* G6 genotype has been reported in sheep from the Tibet Autonomous Region, to the best of our knowledge, this is the first report to describe detection of this genotype in a yak, suggesting the role of sheep and yaks in maintaining CE infection caused by the G6 genotype. This highlights the need to consider the role of other livestock in the transmission and maintenance of the G6 genotype and the contributions to human infection during control programmes. The study also provided additional data on the genetic variation of *E. granulosus* (*s.s.*) in the Tibet Autonomous Region.

## Supplementary information

**Additional file 1: Figure S1.** Sample images of infected lungs (**a**) and liver (**b**) of sheep.

**Additional file 2: Figure S2.** Microscopic images of protoscoleces from hydatid cysts 100× magnification (**a**) and 400× magnification (**b**).

**Additional file 3: Table S1.** List of overlapping primers used in amplifying the complete *Echinococcus canadensis* G6 mitochondrial genome. **Table S2.** Mutation sites of *nad*1 and *nad*5 genes of *Echinococcus granulosus* (*s.s.*) haplotypes found in the Tibet Autonomous Region of China (TAR). **Table S3.** Amino acid changes resulting from *nad*1 and *nad*5 nucleotide mutations.

**Additional file 4: Figure S3.** Bayesian phylogeny of *Echinococcus canadensis* (G6) isolates from Tibet and other G6 (GenBank: MH300930-MH300954, MH300971) and G7 (MH300956-MH300970, MH300972-MH301022) sequences from different countries retrieved from GenBank based on the 12 protein-coding mitochondrial genes. Red indicates isolates from this study [GenBank: MN340038 (YakCHN1) and MN340039 (ShpCHN2)]. *Echinococcus canadensis* G6/G7 reference sequences are from Laurimae et al. [29]. Note that the sequence with GenBank accession MH300971, is the “Gmon” isolate from Mongolia whose genotypic identity and phylogenetic relationship with the G6 and G7 genotypes remain unclear.

## Data Availability

All data supporting the conclusions of this article are included in the article and its additional files. Representative *nad*1 and *nad*5 sequences of *Echinococcus granulosus* (*s.s.*) and the complete mitochondrial genome sequences of *E. canadensis* G6 from the present study were submitted to the GenBank database under the accession numbers MN269986-MN270014 and MN340038-MN340039, respectively.

## Abbreviations

PBS: phosphate buffer solution
PCR: polymerase chain reaction
*cox*1: cytochrome *c* oxidase subunit 1 gene
*nad*1: NADH dehydrogenase subunit 1 gene
*nad*2: NADH dehydrogenase subunit 2 gene
*nad*5: NADH dehydrogenase subunit 5 gene
CE: cystic echinococcosis
MCMC: Markov Chain Monte Carlo
PSRF: potential scale reduction factor
Hd: haplotype diversity
*atp*6: adenosine triphosphatase subunit 6 gene
RNA: ribonucleic acid
