# Supplementary figures and images for: Genetic variation of Echinococcus spp. in yaks and sheep in the Tibet Autonomous Region of China based on mitochondrial DNA
Source: Parasit Vectors. 2019 Dec 27;12:608. doi: 10.1186/s13071-019-3857-1 (PMC6935104; doi:10.1186/s13071-019-3857-1)

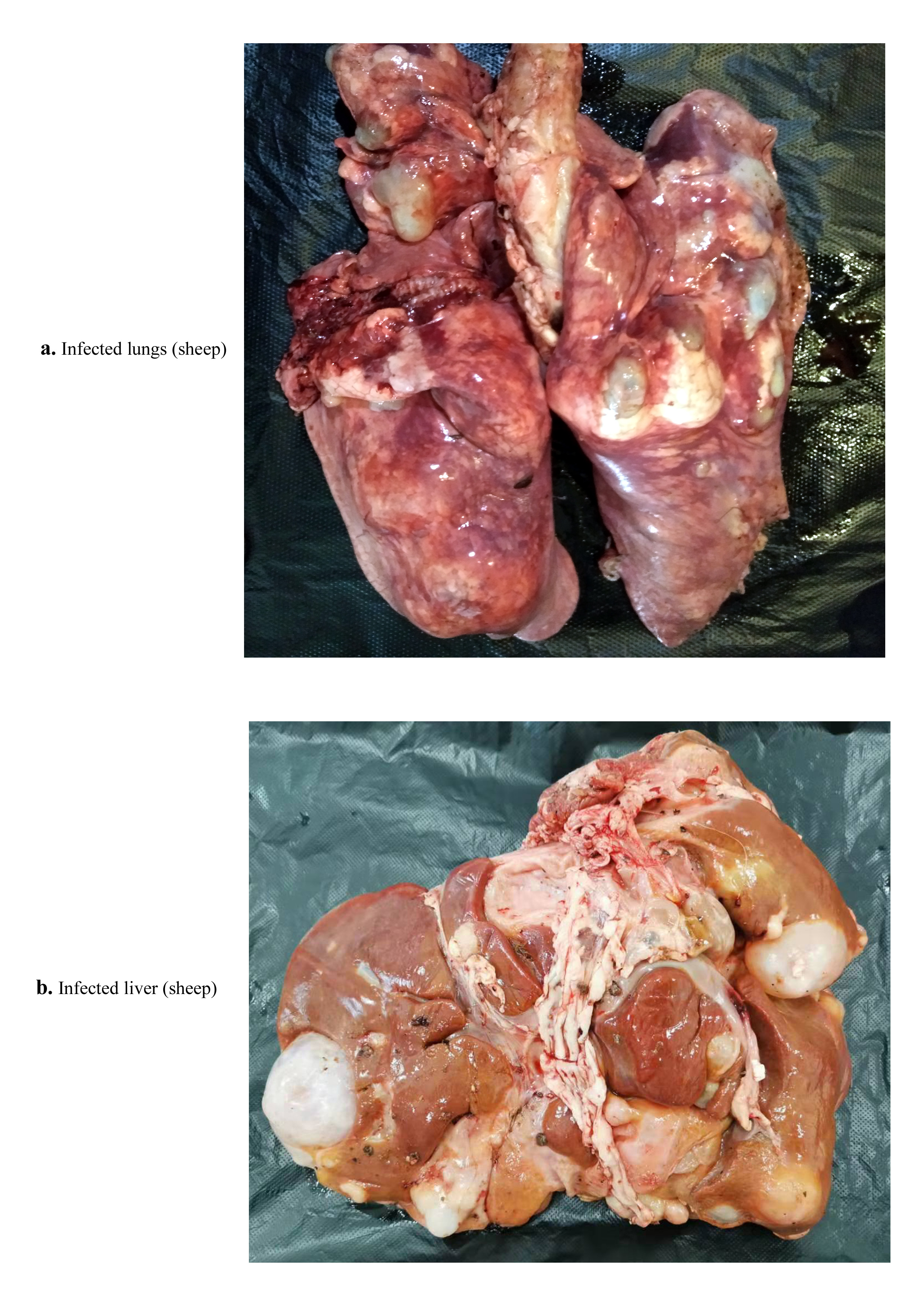

Supplement: Supplementary file 1 — Additional file 1: Figure S1. Sample images of infected lungs (a) and liver (b) of sheep. [file 13071_2019_3857_MOESM1_ESM.jpg]

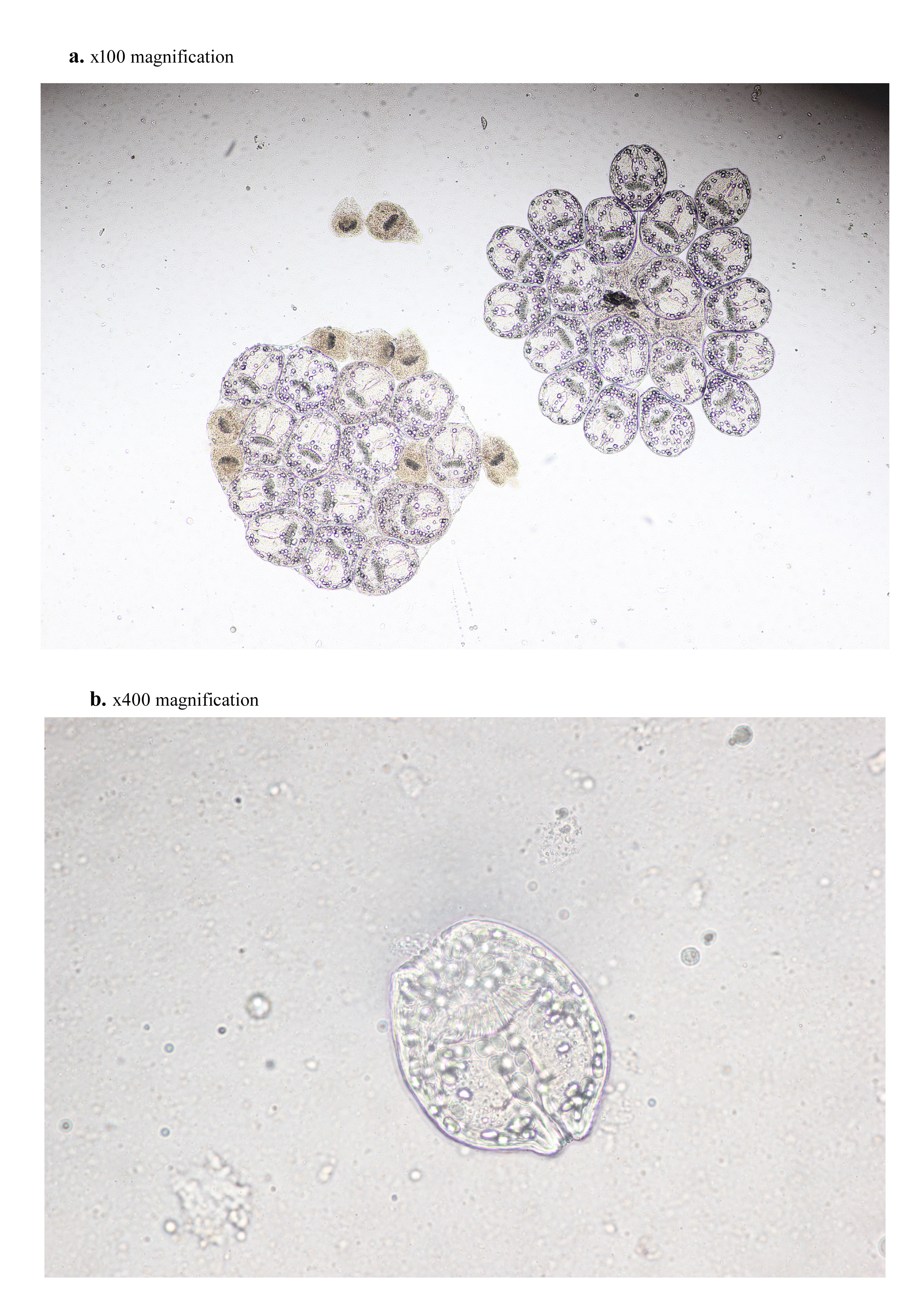

Supplement: Supplementary file 2 — Additional file 2: Figure S2. Microscopic images of protoscoleces from hydatid cysts 100× magnification (a) and 400× magnification (b). [file 13071_2019_3857_MOESM2_ESM.jpg]

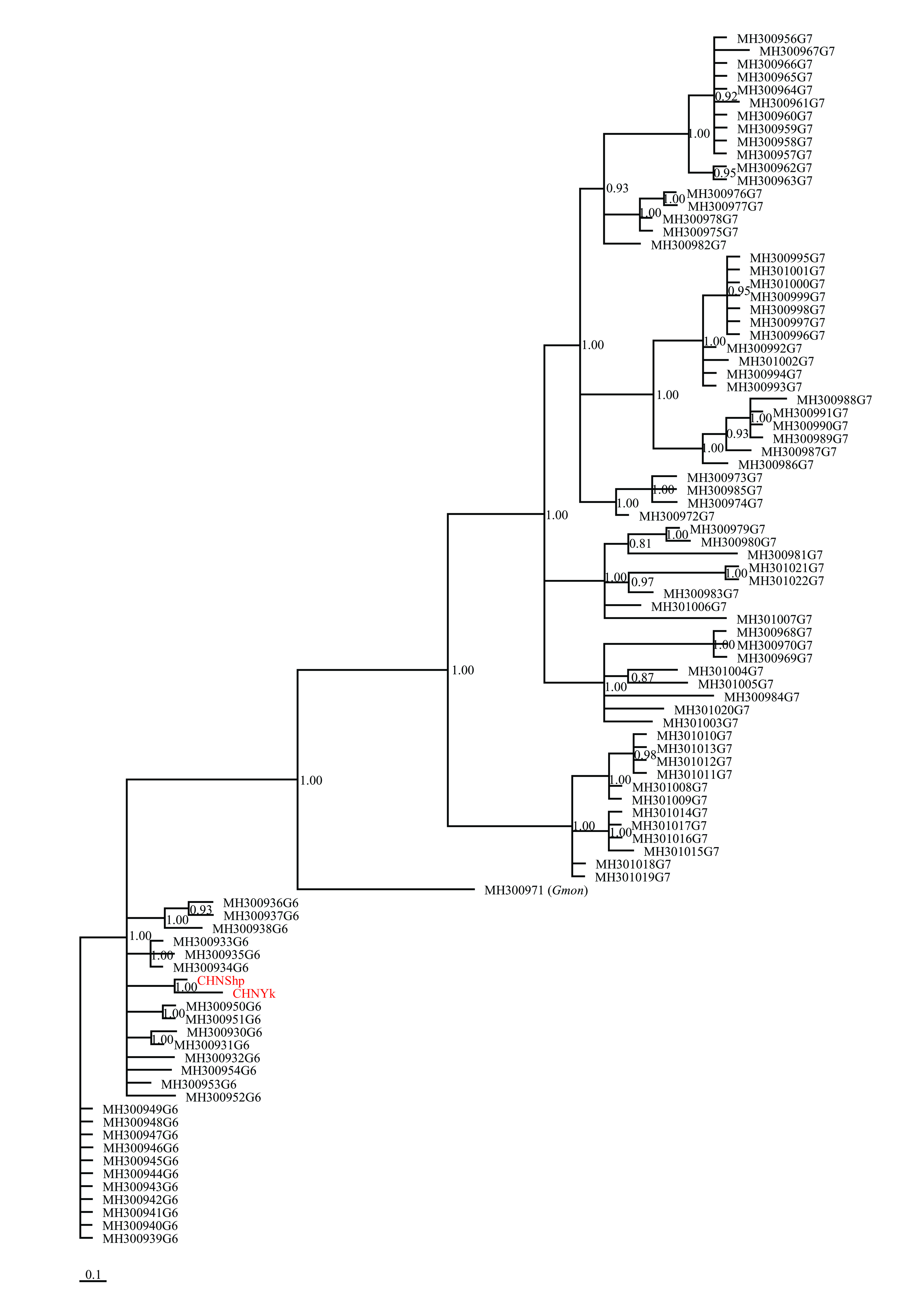

Supplement: Supplementary file 4 — Additional file 4: Figure S3. Bayesian phylogeny of Echinococcus canadensis (G6) isolates from Tibet and other G6 (GenBank: MH300930-MH300954, MH300971) and G7 (MH300956-MH300970, MH300972-MH301022) sequences from different countries retrieved from GenBank based on the 12 protein-coding mitochondrial genes. Red indicates isolates from this study [GenBank: MN340038 (YakCHN1) and MN340039 (ShpCHN2)]. Echinococcus canadensis G6/G7 reference sequences are from Laurimae et al. [29]. Note that the sequence with GenBank accession MH300971, is the “Gmon” isolate from Mongolia whose genotypic identity and phylogenetic relationship with the G6 and G7 genotypes remain unclear. [file 13071_2019_3857_MOESM4_ESM.jpg]
