# Supplementary material for: Genetic variation of Echinococcus spp. in yaks and sheep in the Tibet Autonomous Region of China based on mitochondrial DNA
Source: Parasit Vectors. 2019 Dec 27;12:608. doi: 10.1186/s13071-019-3857-1 (PMC6935104; doi:10.1186/s13071-019-3857-1)
Supplement: Supplementary file 3 — Additional file 3: Table S1. List of overlapping primers used in amplifying the complete Echinococcus canadensis G6 mitochondrial genome. Table S2. Mutation sites of nad1 and nad5 genes of Echinococcus granulosus (s.s.) haplotypes found in the Tibet Autonomous Region of China (TAR). Table S3. Amino acid changes resulting from nad1 and nad5 nucleotide mutations. [file 13071_2019_3857_MOESM3_ESM.docx]

**Additional file 1: Table S1.** List of overlapping primers used in amplifying the complete *Echinococcus canadensis* G6 mitochondrial genome.

| Primer | Primer sequence | Primer position | Amplicon size (bp) |  | Reference |
| --- | --- | --- | --- | --- | --- |
| F1 | TTTGTAAAGATGCCAGAAAA | 244 | 2110 | AB745463 | [15] |
| R1 | AYCTAGATCATTTTTTTGGA | 2356 |  | AB745463 | [15] |
| F1-B* | GTATTCTTTCTGATAGACGAGGTTA | 1778 | 813 | AB208063 | This study |
| R1-B* | CCAAAAACCTACCAAAAATCGCCAC | 2590 |  | AB208063 | This study |
| F2 | GCCCCATATATGTATAGTAT | 2225 | 1962 | AB745463 | [15] |
| R2 | TATACACCGAAGAATAGCAT | 3916 |  | AB745463 | [15] |
| F2-B* | GATAGGTTGCCTGTGGTTGGTCCTA | 3513 | 892 | AB208063 | This study |
| R2-B* | AACATTCTCACACTCGAGTCAACAC | 4404 |  | AB208063 | This study |
| F3 | GATTTRGTGTATTTTCATTCRTA | 3710 | 2508 | AB745463 | [15] |
| R3 | CCAAAACACCCTAACCTAATAT | 6217 |  | AB745463 | [15] |
| F3-B* | GTGTCTGGTTGACTTTGATGATTGTGG | 5831 | 1083 | AB208063 | This study |
| R3-B* | GTAGCGACAGAAGATAAAGATATATG | 6913 |  | AB208063 | This study |
| F4 | ATCGTTTGCCWTATTGTTATAG | 5965 | 2622 | AB745463 | [15] |
| R4 | TAACGGAAAATAAATTCACA | 8586 |  | AB745463 | [15] |
| F5 | TGCTGTTAACTTCAAGAAATGG | 8418 | 1885 | AB745463 | [15] |
| R5 | ACATAACATAATGAAAATGAGC | 10302 |  | AB745463 | [15] |
| F6 | ATATGTTTACTGTTGGGTTRGAT | 10027 | 2352 | AB745463 | [15] |
| R6 | GCAGCACATAGACTTGGCTT | 12379 |  | AB745463 | [15] |
| F7 | CATCTGCGGTTARTCTGTTTTC | 12036 | 2143 | AB745463 | [15] |
| R7 | TAATGCTTAAAACTAACTCATA | 458 |  | AB745463 | [15] |
| F7-B* | GAGGTTGTTGGTTGTTAGTTTGGTTG | 13151 | 1111 | AB208063 | This study |
| R7-B* | CATAAATATCCGCTCTTATGGCCGTA | 540 |  | AB208063 | This study |

*supplementary primers

**Additional file 1: Table S2.** Mutation sites of *nad*1 and *nad*5 genes of *Echinococcus granulosus* *sensu stricto* haplotypes found in Tibet Autonomous Region of China (TAR)

**Table S2a.** *nad*1

| Haplotype | Origin | No. | Variation site | | | | | | | | | | | | | | | |
| --- | --- | --- | --- | --- | --- | --- | --- | --- | --- | --- | --- | --- | --- | --- | --- | --- | --- | --- |
|  |  |  | 43 | 47 | 96 | 108 | 117 | 135 | 178 | 211 | 248 | 296 | 363 | 372 | 378 | 477 | 564 | 649 |
| TB1 | Zhongba, Saga, Dangxiong | 48 | A | G | C | T | C | A | A | A | C | G | T | G | T | T | A | A |
| TB2 | Zhongba, Saga | 9 | - | - | T | - | - | - | - | - | - |  | - | - | - | - | - | - |
| TB3 | Zhongba | 2 | - | - | - | - | T | - | - | - | - | - | - | - | - | - | - | - |
| TB4 | Saga | 2 | - | - | - | - | - | - | - | - | T | - | - | - | - | - | - | - |
| TB5 | Zhongba | 1 | - | - | - | - | - | - | - | - | - | - |  | - | - | - | - | G |
| TB6 | Zhongba, Saga | 3 | - | - | - | - | - | - | - | - | - | - | C | - | - | - | - | - |
| TB7 | Saga | 1 | - | - | - | - | - | - | - | - | - | - | - | - | - | C | - | - |
| TB8 | Zhongba, Saga | 3 | - | - | - | - | - | - | - | G | - | - | - | - | - | - | - | - |
| TB9 | Zhongba, Saga | 6 | - | - | - | - | - | G | G |  | - | - | - | - | - | - | G |  |
| TB10 | Zhongba | 1 | - | - | T | C | - | - | - | - | - | - | - | - | - | - | - | - |
| TB11 | Zhongba | 3 | - | - | - | - | - | - | - | - | - | - | - | A |  | - | - | - |
| TB12 | Zhongba | 1 | - | - | - | - | - | - | - | - | - | - | - | - | G | - | - | - |
| TB13 | Saga | 1 | - | - | - | - | - | - | - | - | - | A | - | - | - | - | - | - |
| TB14 | Saga | 1 | - | C | - | - | - | - | - | - | - | - | C | - | - | - | - | - |
| TB15 | Zhongba | 1 | G | - | T | - | - | - | - | - | - | - | - | - | - | - | - | - |

**Table S2b.** *nad*5

| Haplotype | Origin | No. | Variation site | | | | | | | | | | | | | | | | |
| --- | --- | --- | --- | --- | --- | --- | --- | --- | --- | --- | --- | --- | --- | --- | --- | --- | --- | --- | --- |
|  |  |  | 34 | 39 | 56 | 62 | 124 | 214 | 316 | 325 | 355 | 388 | 404 | 412 | 578 | 619 | 652 | 658 | 661 |
| bTB1 | Zhongba, Saga | 8 | A | G | A | A | G | C | C | T | C | C | G | G | T | A | A | T | G |
| bTB2 | Zhongba, Saga | 34 | - | - | - | - | - | - | - | - | T | - | - | - | - | - | - | - | - |
| bTB3 | Zhongba Dangxiong | 11 | - | - | - | - | - | - | - | - | T | T | - | - | - | G | - | - | - |
| bTB4 | Zhongba, Saga | 14 | - | - | - | - | - | - | - | - | T | - | - | - | C | - | - | - | - |
| bTB5 | Zhongba | 2 | - | - | - | - | - | T | - | - | T | - | - | A | - | - | - | - | - |
| bTB6 | Zhongba | 1 | - | - | - | - | A | - | - | - | T | - | - | - | C | - | - | - | - |
| bTB7 | Zhongba | 1 | G | - | - | - | - | T | - | - | T | - | - | - | - | - | - | - | - |
| bTB8 | Zhongba | 1 | - | - | T | - | - | - | - | - | - | - | - | - | - | - | - | - | - |
| bTB9 | Saga | 1 | - | - | - | - | - | - | - | - | T | - | - | - | - | - | - | C | - |
| bTB10 | Zhongba, Saga | 3 | - | - | - | - | - | - | - | - | T | T | - | - | - | - | - | - | - |
| bTB11 | Saga | 1 | - | - | T | - | - | - | - | - | T | - | - | - | - | - | - | - | - |
| bTB12 | Saga | 2 | - | C | - | G | - | - | T | C | T | - | A | - | - | - | G | - | A |
| bTB13 | Zhongba | 3 | - | C | - | G | - | - | T | - | T | - | A | - | - | - | G | - | A |
| bTB14 | Zhongba | 1 | - | C | - | G | - | - | - | - | T | - | A | - | - | - | G | - | A |

**Additional file 1: Table S3.** Amino acid change resulting from *nad*1 and *nad*5 nucleotide mutation

**Table S3a.** *nad*1

| Haplotype | Variation site | | | | | | | |
| --- | --- | --- | --- | --- | --- | --- | --- | --- |
|  | 15 | 16 | 60 | 71 | 83 | 99 | 188 | 217 |
| TB1 | I | S | 1 | S | A | S | I | S |
| TB2 | - | - | - | - | - | - | - | - |
| TB3 | - | - | - | - | - | - | - | - |
| TB4 | - | - | - | - | V | - | - | - |
| TB5 | - | - | - | - | - | - | - | G |
| TB6 | - | - | - | - | - | - | - | - |
| TB7 | - | - | - | - | - | - | - | - |
| TB8 | - | - | - | G | - | - | - | - |
| TB9 | - | - | V | - | - | - | M | - |
| TB10 | - | - | - | - | - | - | - | - |
| TB11 | - | - | - | - | - | - | - | - |
| TB12 | - | - | - | - | - | - | - | - |
| TB13 | - | - | - | - | - | N | - | - |
| TB14 | - | T | - | - | - | - | - | - |
| TB15 | V | - | - | - | - | - | - | - |

**Table S3b.** *nad*5

| Haplotype | Variation site | | | |
| --- | --- | --- | --- | --- |
|  | 97 | 103 | 105 | 219 |
| bTB1 | S | T | I | G |
| bTB2 | - | - | - | - |
| bTB3 | - | - | - | - |
| bTB4 | - | - | - | - |
| bTB5 | - | - | - | - |
| bTB6 | - | - | - | - |
| bTB7 | - | - | - | - |
| bTB8 | - | S | - | - |
| bTB9 | - | - | - | - |
| bTB10 | - | - | - | - |
| bTB11 | - | S | - | - |
| bTB12 | T | - | V | S |
| bTB13 | T | - | V | S |
| bTB14 | T | - | V | S |
